# The Knowledge, Attitude, and Self-Reported Behaviors of Oncology Physicians Regarding Fertility Preservation in Adult Cancer Patients

**DOI:** 10.1007/s13187-019-01567-6

**Published:** 2019-06-29

**Authors:** Hanfeng Zhang, Guorong Wang, Bin Jiang, Maoqiu Cao, Qinghua Jiang, Li Yin, Bencui Fu, Jian Zhang

**Affiliations:** 1 Department of Radiation Oncology, Sichuan Cancer Hospital & Institute, Sichuan Cancer Center, Radiation Oncology Key Laboratory Of Sichuan Province, School of Medicine, University of Electronic Science and Technology of China, Chengdu, China; 2 Division of Nursing Science, Graduate School of Biomedical and Health Sciences, Hiroshima University, Hiroshima, Japan; 3 Department of Nursing, Sichuan Cancer Hospital & Institute, Sichuan Cancer Center, Radiation Oncology Key Laboratory Of Sichuan Province, School of Medicine, University of Electronic Science and Technology of China, 55, 4th Section of Renmin South Road, Chengdu, 610040 Sichuan Province China

**Keywords:** Fertility preservation, Cancer survivor, Oncology physician, Knowledge, Attitude, Practice behavior

## Abstract

**Abstract:**

There is a growing concern about the fertility preservation for adult cancer patients of reproductive age. Very little literature exists about fertility preservation of cancer survivors in Chinese text. This study is first to describe the knowledge level, attitude, and practice behaviors among physicians concerning fertility preservation in adult cancer patients in China. A cross-sectional survey with 30-item was conducted to assess Chinese oncology physicians’ knowledge, attitude, and behaviors regarding fertility issues. Of 360 oncology physicians, 206 (57.2%) submitted valid questionnaires. With possible overall scores for knowledge and attitude of 9 and 15, respectively, physicians’ responses to the questionnaires were 3.91 ± 1.67 and 12.29 ± 1.23. Only 49.5% of physicians routinely informed their cancer patients of childbearing age about the risk of infertility with cancer treatment. The knowledge score of the men physicians was 2-fold that of the women. Physicians aged 20–29 years were significantly more likely than other age groups to prioritize cancer treatment over fertility concerns. Men physicians were significantly more comfortable than the women discussing fertility preservation issues and cooperating with fertility specialists. The oncology physicians in China had limited knowledge of fertility preservation and rarely discussed these issues with their patients, although their attitude was positive. Results suggest that oncology physicians would welcome an in-house fertility-related training program.

**Key Messages:**

This is the first study to address the topic of fertility preservation as it relates to the care that oncologists provide to cancer patients in China. These results revealed the importance of providing fertility-related training program to oncology physicians. Moreover, this study should provide useful information for other Asian countries, and highlight both the similarities and differences between China and Western countries concerning the reproductive rights of patients. This study should encourage international cooperation with institutions of scientific research and education.

**Electronic supplementary material:**

The online version of this article (10.1007/s13187-019-01567-6) contains supplementary material, which is available to authorized users.

## Introduction

The survival rates of patients with cancer have increased significantly with improvements in detection and treatments, allowing greater attention to their long-term quality of life. The ability to produce children is widely considered to be important for cancer survivors of reproductive age [1].

The fertility of cancer patients can be affected by various factors, including age, cancer type, and cancer treatment [2, 3]. For men, an adverse outcome of cancer treatment may be temporary or permanent azoospermia [4], and women may become unable to produce mature ovum, or suffer premature ovarian failure [2]. In patients whose treatments may lead to infertility, the American Society of Clinical Oncology (ASCO) and the European Society for Medical Oncology (ESMO) have recommended the cryopreservation of sperm and embryos or oocytes in men and women, respectively, as standard strategies for fertility preservation (FP) [1, 5].

Indeed, cancer survivors have a great interest in maintaining fertility, and many prefer to have their own children rather than adopt [6, 7]. Cancer survivors who have preserved their fertility are better ability to cope with their cancer [8], whereas patients without hope of a child often experience anxiety, depression, and grief [9, 10].

Given the importance of FP, international guidelines recommend that physicians should discuss fertility issues with all cancer patients of childbearing age as early as possible [5, 11]. A study based in the UK recommended that patients should be fully informed of the risk of infertility prior to cancer treatments, and alternative treatment strategies should be discussed [12]. Despite these guidelines, many studies have shown that the rate of FP for cancer patients is unsatisfactory and FP is usually not offered [13, 14].

In traditional Chinese culture, continuing the family line is very important. The unmarried infertile woman may remain single, while a married couple is more prone to an unstable marriage and divorce. Hence, in China patients who are infertile experience greater social pressures than do their counterparts in Western countries.

The concerns of Chinese cancer patients who risk damage to their fertility should be given greater attention. However, very little is known of the attitudes and practices of oncology physicians with regard to the preservation of fertility of their patients.

This investigated the current knowledge, attitudes, and practices of Chinese oncologists regarding treatment-related infertility and FP of their patients of reproductive age.

## Methods

The Ethics Committee of Cancer Hospitals approved this study. This survey was conducted from September 2017 to June 2018.

### Subjects and Methods

A cross-sectional survey was conducted to evaluate the knowledge, attitude, and behaviors regarding FP of oncology physicians from three hospitals. The 30-item survey was developed based on previous research conducted in the USA and Britain [15–17], but was modified to be more relevant to Chinese culture. The survey questions were checked and revised by a multidisciplinary team of researchers, oncology physicians, and fertility specialists. The revised survey was piloted with a small group of physicians to test its validity and acceptability. The final version of the survey was the one used in this study.

#### Individual Information

The demographic items in the questionnaire were the following: physician’s gender, age, years working as an oncologist, education, professional title, and specialty in oncology.

#### Knowledge of FP

Physicians were obliged to respond to 9 statements to demonstrate their knowledge of the effect of cancer treatments on fertility, or about FP. Four of these questions assessed knowledge of the effect of cancer treatments on fertility (e.g., “Which chemotherapy drug has a high risk of causing infertility?”). Three questions evaluated basic information regarding FP, which included FP methods, FP organizations, and FP requirements for patients (answering “I know,” or “I do not know”). Physicians were also asked to state if they had sufficient information about FP (“Yes” or “No”), and if they provided comprehensive information regarding FP to patients (“Yes” or “No”). Each question with the right answer or a positive statement (i.e., “I Know” or “Yes”) was scored as 1 point; otherwise, answers were scored as 0 point. The total possible score for overall knowledge was 9.

#### FP Attitudes

All response options to the items were on a three-point Likert scale. One item was used to assess the degree of physician’s level of attention given FP (i.e., not concerned to very concerned). Three items were used to measure the importance that the physician gave to the necessity of discussing fertility with patients (not necessary to very necessary). For example, one item was: “Physicians need to discuss fertility issues with patients.” Physicians were also required to rate the possibility of medical disputes that may be caused by insufficient information about FP (not possible to very possible). Each option of each item was scored from 1 to 3, and the total possible overall attitude score was 15 points.

#### Practice Behaviors

Eight items were used to evaluate physicians’ practice behaviors on a 5-point Likert scale (never to always). Physicians indicated agreement with statements based on their current clinical practice (e.g., “Patients or their families ask me questions concerning reproduction”). A final free-text box was added to allow physicians to comment on the major barriers to mentioning and discussing FP with patients or their families.

### Survey Administration

The survey questionnaire was consisted of an informed consent form, the survey questionnaire, and the e-mail addresses of researchers. Three hundred and sixty oncology physicians in three hospitals were surveyed. Those physicians who were interested in this survey signed the informed consent, finished the questionnaire, and then returned these to the researchers by e-mail.

### Statistical Analysis

Questionnaires with missing data were excluded in the statistical analyses. Data were analyzed using SPSS 19.0 software. All *P* values are two-sided, with a statistical level of significance set at *P* < 0.05. Frequencies and proportions were summarized for demographic characteristics and each survey item. Simple bivariate analyses were used to compare characteristics with knowledge scores and attitude scores. Odds ratios (ORs) and their 95% confidence intervals (CIs) were estimated to compare demographic characteristics, knowledge scores, and attitude scores with current practice behaviors, using ordinal logistic regression. The open-ended data were treated to thematic analysis.

## Results

### Response Rate

In this study, 206 submitted valid questionnaires with no missing data were received, with a response rate of 57.2%.

### Demographic Characteristics

The majority of respondents were men and 30–39 years old. Most of the enrolled physicians were specialists in radiation oncology with a master’s degree and a primary professional title (Table 3).

### Knowledge of FP

The overall total possible score for knowledge for each respondent was 9, but the mean score of the respondents was 3.91 ± 1.67. The rates of knowledge among the respondents regarding fertility damage caused by radiotherapy, targeted therapy, and chemotherapy were 53.4%, 59.2%, and 69.9%, respectively. Furthermore, 60.2% of the participants were unfamiliar with FP methods and FP organizations, and 84.5% had never received any training in cancer treatment-related fertility damage, or FP.

### FP Attitudes of Physicians

The overall total possible score for attitude for each respondent was 15, and the mean score of the participants was 12.29 ± 1.23. Approximately 95.2% of physicians were concerned or extremely concerned about the risk of infertility due to cancer treatment, and 93.2% of them stated that discussions with patients about FP should be conducted more often. About 85% of respondents agreed that providing insufficient FP information to patients could lead to a legal settlement between patients and doctors, and 91.3% thought that guidelines regarding FP should be available in China.

### Practice Behaviors of Physicians

In the clinic, 17.5% of the physicians had never been asked questions concerning reproduction by patients or their families, but only 49.5% of physicians routinely informed their patients of childbearing age about the risk of infertility in cancer treatment. Only 30.1% of physicians always discussed FP with cancer patients of childbearing age; or 25.2% when the patients had previous children. About 22.3% of physicians felt uncomfortable discussing issues related to FP with patients or their families. Instead of choosing lower infertility-damage regimen, 68.9% of physicians altered the treatment with higher survival rate. Only 31.1% of physicians ever consulted a fertility specialist about fertility issues or referred patients to specialists.

### Association Between Knowledge, Attitudes, and Demographic Information

The questionnaires were stratified by knowledge scores (< 5, ≥ 5) and attitude scores (< 12, ≥ 12) to explore associations between these factors and each of the demographic characteristics, using simple bivariate regression. Men are 2.66 times more likely to have high knowledge level of FP as women (OR = 2.66; 95% CI, 1.09 to 7.00) (Table 1). Physicians with a knowledge score < 5 were more likely to report a negative attitude toward FP compared with those with a score ≥ 5 (OR = 0.26; 95% CI, 0.08 to 0.84) (Table 2).

**Table 1:** Logistic regression analysis of factors affecting the fertility-related knowledge of oncologists

	No. of participants (%)	Mean of knowledge	Odds ratio (95% CI)	*p* value
Gender				
Male	126 (61.2)	4.08 ± 1.59	2.66 (1.09–7.00)	0.048*
Female	80 (38.8)	3.64 ± 1.44	–	–
Age (year)				
20–29	66 (32.0)	3.33 ± 1.45	0.08 (0.01–1.08)	0.06
30–39	104 (50.5)	3.57 ± 1.63	0.26 (0.02–3.27)	0.30
40–49	28 (13.6)	3.92 ± 1.69	0.30 (0.02–4.38)	0.38
≥ 50	8 (3.9)	4.75 ± 0.50	–	–
Specialty oncology				
Radiation	78 (37.9)	4.27 ± 1.56	1.42 (0.28–7.12)	0.67
Medical	50 (24.3)	3.56 ± 1.18	0.46 (0.08–2.78)	0.40
Surgical	60 (29.1)	3.65 ± 1.01	0.65 (0.12–3.29)	0.60
Gynecologic	18 (8.7)	3.85 ± 1.74	–	–
Attitude				
< 12	48 (23.3)	3.54 ± 1.19	0.32 (0.10–1.06)	0.06
≥ 12	158 (86.7)	4.03 ± 1.34	–	–

*p < 0.05; *CI*, confidence interval

**Table 2:** Logistic regression analysis of factors affecting the fertility-related attitude of oncologists

	No. of participants (%)	Mean of attitude	Odds ratio (95% CI)	*p* value
Education				
Bachelor’s degree	82 (39.8)	12.07 ± 1.05	0.19 (0.02–1.95)	0.16
Master’s degree	102 (49.5)	12.43 ± 1.25	0.43 (0.04–4.32)	0.47
Doctoral degree	22 (10.7)	12.45 ± 0.82	–	–
Specialty oncology				
Radiation	78 (37.9)	11.90 ± 1.14	0.25 (0.03–2.49)	0.24
Medical	50 (24.3)	11.81 ± 1.12	0.16 (0.02–1.76)	0.14
Surgical	60 (29.1)	11.87 ± 1.16	0.22 (0.02–4.40)	0.22
Gynecologic	18 (8.7)	12.54 ± 1.18	–	–
Knowledge				
< 5	126 (61.2)	11.98 ± 1.36	0.26 (0.08–0.84)	0.02*
≥ 5	80 (38.8)	12.67 ± 0.97	–	–

*p < 0.05; *CI*, confidence interval

### Practice Behaviors of FP Among Physicians

Ordinal logistic regression was used to compare demographic factors, knowledge, and attitude with practice behaviors (Tables 3 and 4). The men physicians were more likely to discuss infertility issues comfortably (OR = 0.41; 95% CI, 0.18 to 0.93) and refer patients to fertility specialists (OR = 14.2; 95% CI, 1.63 to 122) than women physicians. The physicians in the age group 20–29 years were inclined to prioritize the cancer treatment over fertility considerations (OR = 0.05; 95% CI, 0.01 to 0.82) and failed to inform patients about FP before cancer treatment (OR = 0.09; 95% CI, 0.01 to 0.778). The oncologists with bachelor’s degree and master’s degree were more likely to discuss specific FP methods (OR = 12.9; 95% CI, 2.78 to 60.10; OR = 15.4; 95% CI, 3.54 to 66.60) and infertility issues with patients (OR = 0.12; 95% CI, 0.04 to 0.97; OR = 0.12; 95% CI, 0.02 to 0.66). Compared with gynecologic physician, surgical physician was less likely to inform the patients about the FP issues (OR = 0.18; 95% CI, 0.03 to 0.92) and discuss reproductive arrangements (OR = 0.08; 95% CI, 0.02 to 0.40). In addition, a negative attitude toward FP was significant factors associated with infrequently informing patients about FP and asking their plans for future pregnancies (OR = 0.36; 95% CI, 0.14 to 0.96; OR = 0.38; 95% CI, 0.15 to 0.97; OR = 0.19; 95% CI, 0.07 to 0.51).

**Table 3:** Demographics of participants, *n* (%)

Total subjects		206 (100)
Gender	Male	126 (61.2)
	Female	80 (38.8)
Age (year)	20–29	66 (32.0)
	30–39	104 (50.5)
	40–49	28 (13.6)
	≥ 50	8 (3.9)
Education	Bachelor’s degree	82 (39.8)
	Master’s degree	102 (49.5)
	Doctoral degree	22 (10.7)
Professional title	Primary	104 (50.5)
	Intermediate	74 (35.9)
	Senior	28 (13.6)
Specialty oncology	Radiation	78 (37.9)
	Medical	50 (24.3)
	Surgical	60 (29.1)
	Gynecologic	18 (8.7)

**Table 4:** Logistic regression analysis of factors affecting the fertility-related practice behaviors of oncologists

	Gender		Age				Education			Specialty oncology				Knowledge		Attitude	
	Male	Female	20–29	30–39	40–49	≥ 50	Bachelor’ degree	Master’ degree	Doctoral degree	Radiation	Medical	Surgical	Gynecologic	< 5	≥ 5	< 12	≥ 12
B1																	
OR	0.22	1.0	11.7	15.2	22.2*	1.0	9.16*	4.69*	1.0	3.2	2.5	2.3	1.0	0.42	1.0	0.22	1.0
95% CI					1.90–258		1.86–45.0	1.08–20.2									
B2																	
OR	0.41	1.0	5.4	7.8	3.3	1.0	2.54	3.98*	1.0	6.2	3.6	0.18*	1.0	0.16	1.0	0.36*	1.0
95% CI								1.01–15.80				0.03–0.92				0.14–0.96	
B3																	
OR	0.26**	1.0	2.7	5.6	3.4	1.0	5.25	4.06*	1.0	2.7	2.3	0.08*	1.0	0.55	1.0	0.38*	1.0
95% CI	0.12-0.60							1.06–15.6				0.02–0.40				0.15–0.97	
B4																	
OR	0.52	1.0	8.9	10.2	7.3	1.0	12.9*	15.4*	1.0	3.37	2.13	2.05	1.0	0.16*	1.0	0.19*	1.0
95% CI							2.78–60.10	3.54–66.59						0.09–0.79		0.07–0.51	
B5																	
OR	0.41*	1.0	0.78	0.35	0.26	1.0	0.12*	0.12*	1.0	0.36	0.42	0.18	1.0	0.33	1.0	0.78	1.0
95% CI	0.18–0.93						0.04–0.97	0.02–0.66									
B6																	
OR	0.67	1.0	0.09*	0.54	0.87	1.0	3.6	6.5	1.0	2.78	3.16	1.12	1.0	0.15	1.0	0.16	1.0
95% CI			0.01–0.78														
B7																	
OR	0.54	1.0	0.05*	0.42	0.13	1.0	4.4	6.2	1.0	1.56	2.18	1.09	1.0	0.18	1.0	0.34	1.0
95% CI			0.01–0.82														
B8																	
OR	14.2*	1.0	12.6	11.5	8.3	1.0	3.1	4.8	1.0	0.78	0.65	0.23	1.0	0.17	1.0	0.21	1.0
95% CI	1.63–122																

*p < 0.05; **p < 0.01

*OR*, odds ratio; *CI*, confidence interval

B1 = Patients or their families ask me questions concerning reproduction;

B2 = I regularly inform cancer patients of childbearing age about the risk of infertility from cancer treatment;

B3 = For patients with children, I ask about their plans for future pregnancies and inform them of the risk of infertility;

B4 = I discuss fertility preservation methods with patients who have the desire to have a baby;

B5 = I feel uncomfortable discussing infertility issues with patients or their families;

B6 = I check with patients about the importance of their fertility;

B7 = I choose the lower infertility-damage cancer treatment regimen even if it has a lower survival rate;

B8 = I consult a fertility specialist about fertility issues in my patients and refer the patients to a specialist;

### Barriers for Discussion with Patients Concerning FP

Poor prognosis was regarded as a major barrier for talking about FP issues with patients (66.9%). Other major reasons that hindered discussion by oncology physicians with their patients were the following: limited knowledge of the physician about FP (43.6%), marital status of patients (30.5%), time constraints (16.5%), the economic status of the patient (15.4%), a delay in cancer treatment associated with FP (13.5%), and ethical issues (8.1%).

## Discussion

This study investigated the knowledge, attitudes, and practices of oncologists at a cancer hospital in China regarding reproductive issues in cancer patients of reproductive age. A 30-item survey was adapted specific to Chinese culture and 206 (57.2%) physicians responded with completed questionnaires. The major findings were that knowledge of how cancer treatments affect patients’ fertility, or how fertility may be preserved, was very low in these physicians, but their attitudes toward change in these matters were positive. Only 49.5% of the physicians routinely discussed reproductive issues with their patients. Men physicians were considerably better informed about these issues than the women, more willing to discuss them with their patients, and more willing to cooperate with fertility specialists. Compared with older age groups, younger physicians (aged 20–29 years) were more likely to prioritize cancer treatments over preserving fertility in their patients. A high majority of physicians understood the need for consulting patients regarding preserving fertility, but also believed that supportive guidelines for addressing these issues should be available to them.

The knowledge score of the oncology physicians regarding FP was unsatisfactory, being only 3.91 out of a possible 9. This was surprising that more than half of the oncology physicians were not familiar with the fertility damage that is associated with anticancer treatment. In addition, fewer than 40% of physicians were aware of FP methods and FP organizations although an FP organization was just 5.4 km away from the hospital. Physicians with the lowest knowledge scores had the least positive attitudes regarding FP issues (OR = 0.26), but nevertheless, the majority of physicians held positive attitudes toward FP. This is similar to the knowledge and attitudes of physicians reported from other countries [16, 17]. The discrepancy between physicians’ positive attitudes but low level of knowledge concerning FP seems to indicate a lack of fertility-related training. In the present study, more than 80% of participants had never participated in any FP-related training program. Nevertheless, it is encouraging that the Asian Society for Fertility Preservation (ASFP) has been founded, and their first Asian congress on FP took place in Vietnam in 2016. This was a big step toward improving FP-related training and promoting information exchange among oncology professionals in Asia. There is no doubt that more fertility-related education will be developed and provided to oncologists in China and other Asian countries in the near future. In addition to continuing education, we also recommend that the importance of fertility protection should be reinforced in medical education by teachers. Given that the mean knowledge score of female oncologists was only 3.64, and the knowledge score of the male physicians was 2-fold that of the female physicians (OR = 2.66, *p* < 0.05), female oncologists therefore should pay more attention to increase their fertility-related knowledge to support the implementation of comprehensive and equitable FP practices.

As for the practice behaviors regarding FP, the percentages of oncologists who discuss fertility issues with their cancer patients in Saudi Arabia, Japan, and British were reportedly 42%, 42.7%, and 97%, respectively [17–19]. By contrast, 49.5% of physicians in the present study routinely consulted cancer patients of childbearing age about FP issues and just 30.1% discussed FP procedure, which suggest that discussion of fertility preservation in China remains inadequate. Results also indicated that male oncologists were more likely to feel comfortable discussing FP problems and willing to refer patients to fertility specialists (OR = 0.41, *p* < 0.05; OR = 14.2, *p* < 0.05). In contrast, one study revealed that the likelihood of referring was associated with the gender of the practicing oncologists, with female oncologists more likely to refer [15]. However, a newly published research showed that no statistically significant differences were observed between male and female oncologists regarding the referral behaviors [20]. These differences could be due to the various cultural contexts in which the surveys took place. Moreover, the number of women in this sample was quite low compared with the men. Further studies with a greater proportion of women should be conducted before any convincing conclusions can be made in China.

Currently, there are three academic levels of educational background (bachelor’s, master’s, and doctoral) for Chinese oncologists who are qualified to prescribe and may discuss the FP issues with patients. Some interesting findings also have been discovered that the oncologists with bachelor’s degree or master’s degree were more likely to discuss specific FP methods (OR = 12.9, *p* < 0.05; OR = 15.4, *p* < 0.05) and feel more willing to mention infertility issues (OR = 0.12, *p* < 0.05; OR = 0.12, *p* < 0.05) than physicians with doctoral degree. Meanwhile, compared with gynecologists, surgical physicians were less likely to inform the patients about the FP issues (OR = 0.18, *p* < 0.05; OR = 0.08, *p* < 0.05). It is very vital to acknowledge that the failure to inform patients adequately about FP can lead to lawsuits from some patients, and this was known by 85% of physicians. The possibility of legal settlements was also recognized in another study [21]. Given that there is presently a rather tense relationship between patients and health care providers in China, most oncologists understand that informing and discussing FP with cancer patients should be improved. Therefore, oncologists with doctoral degree or the surgical physicians are particularly encouraged to enhance professional practices in relation to FP and prevent loss of cancer patients’ fertility. This should ensure the legitimate rights of patients and also protect the physicians themselves. In addition, 91.3% of physicians in this medical specialty thought it necessary to issue guidelines regarding FP in China, just as in other countries.

It is worth mentioning that only 25.2% of respondents in this study were concerned about FP when patients already had children. However, according to one survey, women with children may be confused about their reproductive plans, because they want children with a new partner or consider their family incomplete [22]. In October 2015, the government of China approved a new child policy as a strategy to compensate for an aging population, and each couple is now allowed two children. The new policy means that every Chinese person, including cancer patients, should have the choice to have another baby. Therefore, it should be guaranteed that every patient of childbearing age should be equally provided with sufficient information regarding FP, whether they already have children or not.

International guidelines emphasize that oncologists should be well prepared to discuss possible PF options, or refer appropriate patients to fertility specialists [1, 5]. However, the results of the present study show that more than half of the physicians were reluctant to talk with a fertility specialist about fertility issues, or refer patients to specialists. This is similar to other reports [15, 21]. Previous studies found that some physicians would decline to abide by the guidelines if the patient could not afford the practice [23]. In addition to considering the economic status of patients, low rates of referral may be because the oncologist considers the cancer treatment a greater priority. It is a tough call for the consulting oncologist whether he/she should choose to provide a better care and focus on the cancer treatment or offer the possibility to conceive instead. Nearly 70% of the participants in the current study reported that achieving longer survival was more important than maintaining fertility. This attitude was especially prevalent in oncologists in the 20–29 year age range (OR = 0.09, *p* < 0.05; OR = 0.04, *p* < 0.05). However, at least one study showed that some patients were willing to sacrifice a little survival in the interest of better fertility outcomes, and preferred to consider less aggressive cancer regimes [24]. Hence, we suggest that a final choice of FP should be left to the informed patient, rather than the will of the physician. If patients do not intend to have children, anticancer treatments can be carried out according to plans. However, if patients have the desire to preserve their fertility, we recommend oncologists, fertility specialists, and psychologists to work together and have a detailed discussion about FP with them, including provide professional treatment protocols, introduce the procedure of FP and reliable referral pathways, discuss the risks and also the benefits of every protocol, and provide psychological support for patients and their families. Only upon the patient’s consent, the best possible treatment be then given, preferably on the basis of preserving the patient’s fertility. Moreover, it is worth noting that young oncologists should be encouraged to assist patients to understand FP options and weigh up choices by delivering oncofertility services according to international guidelines.

The present study identified many barriers to discussions concerning FP by oncologists with their patients. The major bottleneck for oncologists to initiate FP discussion with cancer patients was a poor prognosis for the patient. This reflects the Chinese culture of the physician, in which good news is given rather than bad. Thus, it is difficult for physicians to disclose a poor prognosis, especially for a Chinese oncologist to discuss reproductive arrangements when the patient has short survival time. However, to some extent, we need to acknowledge that discussing FP issues is an easy way to avoid talking about the coming death of the patients. In addition, patients who know the available FP options often have more hope and more concern for their future. Therefore, more discussion regarding FP between oncologists and patients should be encouraged, and some strategies for improving discussion skills for oncologists are ought to be provided, including how to give difficult news to patients and how to facilitate discussion about FP with advanced cancer patients.

Concomitant with the above, other barriers that influenced discussions included time constraints, a possible delay in cancer treatment, lack of knowledge or ethical concerns of the physician, and the marital status, or limited economic means of the patient [25]. Future efforts in China should be directed toward promoting knowledge of FP for oncologists by providing systematic training programs, an insurance system that would cover the expense of FP technologies, and consumer laws regarding FP. When time constraints are a factor in informing patients of FP and treatment options, we suggest that such consultations could be covered by multiple disciplines, including oncology physicians, nurses, social workers, and other health care providers, and especially fertility specialists. It is worth noting that the time required for FP is different based on the cancer type and patients’ physical condition, so some physicians refuse to discuss the FP issues with the perception that patients could not delay treatment to pursue options when the patients requiring immediate cancer treatment [15, 18]. On the contrary, one study showed that some oncologists who believed that discussion should not be held if patients had an exceedingly aggressive form of cancer and required immediate treatment were more likely to discuss FP issues [20]. Thus, it can be seen that preventing a delay in an urgent treatment might not be a barrier for discussing. In a word, we strongly suggested discussing about FP issues with patients in reproductive age, no matter what situation it is in.

### Limitations of the Study

In general, physicians’ response to surveys is low, reportedly 15% and 37.6% in the USA and British, respectively [16, 17], and 57.2% in the present study. While the latter is slightly higher than that of previous studies, this suggests that a large proportion of oncologists in China are not interested in or familiar with this topic. It is also important to acknowledge that this study explored the attitudes and practices toward FP of physicians who treat only adult cancer patients. Thus the opinions of pediatric physicians, oncology nurses, and cancer survivors were not included. It is necessary to initiate multicenter studies across China to understand comprehensively the current situation regarding FP, and such studies should include oncologists, nurses, and cancer survivors.

## Conclusions

Although the Chinese oncologists in this study had limited knowledge of FP and unsatisfactory practice behaviors by self-report, their attitude toward FP in cancer patients of reproductive age was generally positive. This suggests the need for training programs conducted by multidisciplinary teams, the development of a standard guideline for physicians, a comprehensive law, and an insurance system that covers FP in China. This is the first study to address the topic of FP as it relates to the care that oncologists provide to cancer patients in China. These results should provide useful information for other Asian countries and highlight both the similarities and differences between China and Western countries concerning the reproductive rights of patients. This study should encourage international cooperation with institutions of scientific research and education.

### Lessons for Practice

The oncology physicians in China had limited knowledge of fertility preservation and rarely discussed these issues with their patients, although their attitude was positive. A systemic fertility-related training program should be organized for physicians to advance knowledge and facilitate practice regarding fertility preservation. A multidisciplinary team with fertility specialists, a sound insurance system, a comprehensive law, and a guideline should be developed for promoting appropriate use of fertility preservation in cancer survivors in China in the near future.

## Electronic supplementary material

ESM 1 (DOC 36 kb)
