# Supplementary material for: The Knowledge, Attitude, and Self-Reported Behaviors of Oncology Physicians Regarding Fertility Preservation in Adult Cancer Patients
Source: J Cancer Educ. 2019 Jun 29;35(6):1119–27. doi: 10.1007/s13187-019-01567-6 (PMC7679324; doi:10.1007/s13187-019-01567-6)
Supplement: Supplementary file 1 — (DOC 36 kb) [file 13187_2019_1567_MOESM1_ESM.doc]

**Supp. Questions of each category (knowledge, attitude and practice) in the questionnaire**

Questions of knowledge in questionnaire (K=knowledge)

K1. Which chemotherapy drug has a high risk of causing infertility?

K2. Which therapy can damage human fertility?

K3. Have you ever received any training on fertility preservation?

K4. How long after the cancer treatment can a patient procreate?

K5. Do you know any organizations where you can preserve your fertility?

K6. Do you know any methods for fertility preservation?

K7. Do you know which patients are eligible for fertility preservation?

K8. Do you have sufficient information about FP?

K9. Do you often provide comprehensive information regarding FP to patients?

Questions of attitude in questionnaire (A=attitude)

A1. Are you concern about the risk of infertility due to cancer treatment?

A2. Do you think it is necessary for oncologists to be trained on the reproductive toxicity and fertility preservation related to cancer treatment?

A3. Do you think it is necessary for oncologists to inform and talk to the cancer patients about reproductive toxicity and fertility preservation?

A4. Do you think it is necessary to develop some guidelines regarding fertility preservation in China?

A5. Do you think it is possible to cause medical disputes if medical staffs provide insufficient information about fertility preservation for patients?

Questions of practice behavior in questionnaire (B=behavior)

B1. Patients or their families ask me questions concerning reproduction

B2. I regularly inform cancer patients of childbearing age about the risk of infertility from cancer treatment

B3. ­­­For patients with children, I ask about their plans for future pregnancies and inform them of the risk of infertility

B4. I discuss fertility preservation methods with patients who have the desire to have a baby

B5. I feel uncomfortable discussing infertility issues with patients or their families

B6. I check with patients about the importance of their fertility

B7. I choose the lower infertility-damage cancer treatment regimen even if it has a lower survival rate

B8. I consult a fertility specialist about fertility issues in my patients and refer the patients to a specialist
